# As(III) Removal from Aqueous Solution by Calcium Titanate Nanoparticles Prepared by the Sol Gel Method

**DOI:** 10.3390/nano9050733

**Published:** 2019-05-13

**Authors:** Rocío Tamayo, Rodrigo Espinoza-González, Francisco Gracia, Ubirajara Pereira Rodrigues-Filho, Marcos Flores, Elisban Sacari

**Affiliations:** 1 LabMAM, Depto. de Ingeniería Química Biotecnología y Materiales, FCFM, Universidad de Chile, Av. Beauchef 851, Santiago 8370456, Chile; rocio.tamayo@ing.uchile.cl (R.T.); fgracia@ing.uchile.cl (F.G.); 2 Grupo de Química de Materiais Híbridos e Hinorgânicos, Instituto de Química de Sao Carlos, Universidade de São Paulo, 13563-120 Sa͂o Carlos, SP, Brazil; ubirajara@usp.br; 3 Laboratorio de Superficies, Depto. de Física, FCFM, Universidad de Chile, Av. Blanco Encalada 2008, Santiago 8370449, Chile; mflorescarra@ing.uchile.cl; 4 Laboratorio de Nanomateriales, Facultad de Ingeniería, Universidad Nacional Jorge Basadre Grohmann, Av. Miraflores s/n, Tacna 23003, Peru; esacaris@unjbg.edu.pe

**Keywords:** arsenic adsorption, sol-gel technique, calcium titanate, nanoparticles, adsorption kinetic

## Abstract

Arsenic (As) contamination of water is a serious problem in developing countries. In water streams, arsenic can be as As(V) and As(III), the latter being the most toxic species. In this work, an innovative adsorbent based on CaTiO_3_ nanoparticles (CTO) was prepared by the sol-gel technique for the removal of As(III) from aqueous solution. X-ray diffraction of the CTO nanoparticles powders confirmed the CTO phase. Transmission electron microscopy observations indicated an average particle size of 27 nm, while energy dispersive X-ray spectroscopy analysis showed the presence of Ca, Ti, and O in the expected stoichiometric amounts. The surface specific area measured by Brunauer, Emmett, and Teller (BET) isotherm was 43.9 m^2^/g, whereas the isoelectric point determined by Zeta Potential measurements was at pH 3.5. Batch adsorption experiments were used to study the effect of pH on the equilibrium adsorption of As(III), using an arsenite solution with 15 mg/L as initial concentration. The highest removal was achieved at pH 3, reaching an efficiency of up to 73%, determined by X-ray fluorescence from the residual As(III) in the solution. Time dependent adsorption experiments at different pHs exhibited a pseudo-second order kinetics with an equilibrium adsorption capacity of 11.12 mg/g at pH 3. Moreover, CTO nanoparticles were regenerated and evaluated for four cycles, decreasing their arsenic removal efficiency by 10% without affecting their chemical structure. X-ray photoelectron spectroscopy analysis of the CTO surface after removal experiments, showed that arsenic was present as As(III) and partially oxidized to As(V).

## 1. Introduction

Water is one of the fundamental factors for the development of humanity. The contamination of water sources with arsenic (As) is a serious worldwide problem. It is estimated that between 60 and 100 million people in the world are exposed to the presence of As in drinking water, in concentrations harmful to health. It has been reported that at least 21 countries have high concentrations of As in their groundwater [1,2]. The most affected areas in the world are South-East Asia, in countries such as Bangladesh, India, Nepal, Taiwan and Vietnam, where concentrations of As that exceed 1 mg/L have been reported [3]. The World Health Organization (WHO) sets as maximum permissible limit 10 μg/L of As in drinking water [4].

It is known that in Latin America that the problem of As pollution is present in more than 14 countries, such as Argentina, Bolivia, Brazil, Chile, Colombia, Cuba, Ecuador, El Salvador, Guatemala, Honduras, Mexico, Nicaragua, Peru, and Uruguay [5]. Arriaza et al. carried out studies on samples of mummies belonging to the Chinchorro culture, who lived between the cities of Ilo in Southern Peru and Antofagasta in Chile 7000 years ago, reporting that they suffered from arsenic, which could be caused by ingestion of food and water contaminated with As [6]. Waters contaminated with As are a hazard to humans, and chronic exposure to contaminated water intake causes different diseases. Some studies indicate that As could be associated with skin cancer and internal organs [7].

In water streams, arsenic chemical species are arsenate (As(V)) and arsenite (As(III)) [8]. As(III) is the major species under reducing environmental conditions, as well as the most toxic species [9,10]. As(V) is negatively charged in the pH range of 2.5–12, while As(III) is negatively charged above pH 9 [11].Therefore, arsenite or arsenate sorption from aqueous solution is strongly dependent on pH and surface stability constants whenever inner-sphere complex formation comes into play [12,13].

The removal of As has been improved with the advances of technology. The most used processes for removal of As are coagulation, oxidation, precipitation, adsorption with different materials, resins for ion exchange, and membrane technology as conventional methods. At large scale the most employed are the coagulation methods with Al and Fe salts although combined with other processes as filtration and oxidation [2]. Hu et al. established an optimal pH between 5 and 7 in the study of the effects of aluminum speciation during the removal of As(V) in coagulation processes [14]. Lacasa et al. [15] reported the removal of 10 μg/dm^3^ of As using an electrocoagulation process with electrodes of Fe and Al; while Mohora et al. [16] obtained 93% removal of As using a continuous electrocoagulation/flocculation process.

The adsorption of As(III) by nanocomposites of Cu-chitosan/nano-Al_2_O_3_ has also been studied; and the high adsorption capacity and high initial velocity of this nanocomposite were demonstrated [17]. Hlavay et al. studied the superficial properties of alumina covered with Fe(OH)_2_ for which the adsorption capacity is a function of the pH. They obtained a selective and efficient adsorbent for arsenite and arsenate ions [18]. Activated carbon is a widely used material in adsorption of arsenic. Vitela et al. [19] investigated arsenic adsorption by activated carbon modified with Fe nanoparticles and reached a maximum capacity of As(V) of 1.25 mg/g, that decreases by 32% when the pH increases from 6 to 8. Mesoporous activated carbon for the adsorption of As(III)/As(V) has also been studied reaching a maximum removal of 1.491 and 1.760 mg/g [20]. Complex absorbents such as Fe–Mn–Ce ternary oxide–biochar composites have been recently reported by Liu et al. [21] for the removal of As(III); which exhibited a maximum sorption capacity of 8.47 mg/g and the greatest adsorption at pH 3. Similarly, good adsorption results have been obtained for metal–organic framework–graphene oxide (MOF-GO) nanocomposites. Chowdhury et al. [22] showed high adsorption capacity of MOF-GO materials but with the maximum capacity at pH 11.

As is mainly found as compounds of As(V) and As(III). The former is present in surface waters and the second in groundwater. It is known that As(III) is more toxic and difficult to remove from water than As(V), so a strategy for removal is first to oxidize the As(III) to As(V) to achieve a more effective removal [9]. This implies that the treatment methods used today have several stages that lead to higher costs.

Due to the above, there is a motivation to search for new alternatives for the removal of As, that allow the treatment of removal in a single stage. This work proposes the use of nanoparticles of perovskite type CaTiO_3_ (CTO) obtained by the sol-gel methods, for the adsorbent function of As(III), which has been little studied with this type of perovskite. In addition, it will allow us to look for the generation of a new system that facilitates the removal of As (III) using a simple ceramic prepared from earth abundant materials.

## 2. Materials and Methods

### 2.1. Synthesis of CaTiO_3_ Nanopowder

**Figure 1:**
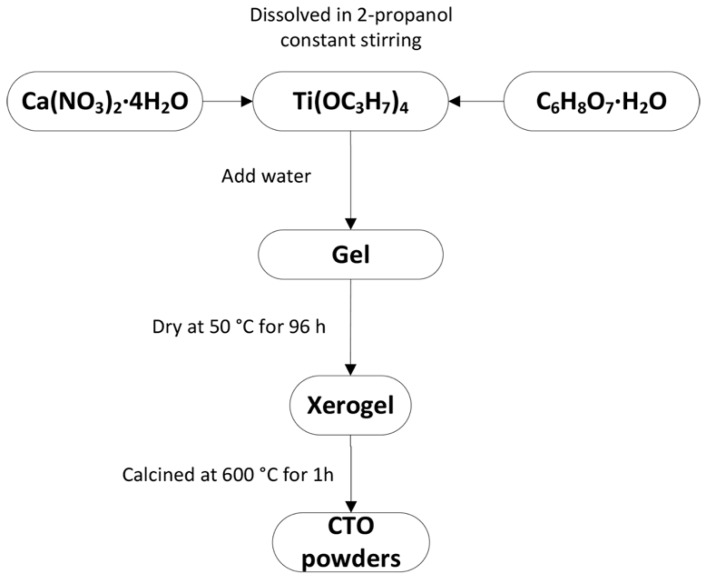
Flowchart for CaTiO_3_ nanoparticles (CTO) preparation procedure.

Pure CaTiO_3_ (CTO) perovskite nanoparticles were prepared by the sol-gel method. In this synthesis, titanium (IV) isopropoxide Ti(OC_3_H_7_)_4_ (97% Sigma-Aldrich, St. Louis, MO, USA ), calcium (II) nitrate tetrahydrate Ca(NO_3_)_2_∙4H_2_O (99% Sigma-Aldrich, St. Louis, MO, USA), 2-propanol (EMSURE®, Merk, Darmstadt, Germany), and citric acid monohydrate C_6_H_8_O_7_∙H_2_O (≥99% Sigma-Aldrich, St. Louis, MO, USA), were used as starting materials. All the chemicals were of analytical grade and no further purification was performed. The synthesis steps are the following (Figure 1): a) Ti(OC_3_H_7_)_4_, Ca(NO_3_)_2_∙4H_2_O and citric acid were weighed accurately according to the stoichiometric composition, and dissolved separately in 2-propanol, under vigorous stirring for 30 min; b) once the solution of Ca(NO_3_)_2_∙4H_2_O was added drop by drop to the solution of Ti(OC_3_H_7_)_4_), the solution of citric acid was likewise added drop by drop to obtain a CTO precursor solution; c) this last solution was stirred vigorously for 30 min at room temperature while deionized water was slowly added until the gel was formed; d) then, the gel was dried at 50 °C for 96 h. The resultant powder (xerogel) was ground in an agate mortar for 5 min and finally calcined at 600 °C over 1 h in air to obtain CTO powder.

### 2.2. Characterization

Thermal properties of the dry gel precursor were determined using a thermogravimetric analyzer TA instrument, model TGA Q-50 and differential scanning calorimeter DSC Q-20 (TA instrument, New Castle, DE, USA), under nitrogen atmosphere and at a heating rate of 5 °C/min. The obtained powders were characterized by X-ray diffraction (XRD) in a D8 Bruker (Billerica, MA, USA) diffractometer using CuKα radiation (λ = 1.5418 Å). The morphology and microstructure of the samples were studied by transmission electron microscopy (TEM) in a FEI Tecnai F20 FEG-S/TEM (Eindhoven, The Netherlands) microscope operated at 200 kV, with EDAX detector for energy dispersive X-ray spectroscopy (EDX). The specific surface area of the powders was measured by nitrogen adsorption isotherm using the Brunauer, Emmett and Teller (BET) equation in a Micromeritics ASAP 2010 (Norcross, GA, USA) apparatus at −196 °C. The point of zero charge (pH_pzc_) was measured employing a ZSNano Zetasizer instrument (ZEN 3600, Malvern, Worcestershire, UK). A solution of KCl with a concentration of 0.003 mol/L was prepared, in which the CTO sample was added; to this solution of KCl + CTO, HCl and KOH were added to vary the pH.

### 2.3. Batch Adsorption Experiments

Different sets of 100 mL solutions containing As(III) were prepared using sodium arsenite (NaAsO_2_) with an initial concentration of 15 mg L^−1^ and different pHs (1 to 11), and 100 mg CTO was added. This mixture was stirred and 2 mL aliquots were taken at 6, 12, 18, 24, 30, 40, 60, and 80 min. The total As concentration in the aliquots was determined by using X-ray fluorescence analysis using a benchmark MiniPal4 (PANalytical, Almedo, The Netherlands) spectrometer in energy dispersive mode. The semi-quantitative determination of arsenic removal was performed using the standard less analysis package Omnian (PANalytical). All the measurements were acquired after a total measurement time of 840 s and were done in triplicate. A similar process was employed to evaluate the potential dissolution of CTO under acidic conditions at pH 1, 2, and 3. In this case, the aliquots were centrifuged to separate solids and the residual liquid was analyzed by XRF.

On the other hand, the final solutions after 80 min. of the adsorption experiments were centrifuged of at 5000 rpm on 10 min to recover the solids of CTO nanoparticles. The solids were dried at 115 °C over 3 h to study their superficial chemical composition after adsorption, which was analyzed by means of the X-ray photoelectron spectroscopy technique (XPS). This was performed using an XPS–Auger PerkinElmer spectrometer model PHI 1257 (PerkinElmer Corporation, Eden Prairie, MN, USA), with an X-ray source of Kα radiation from an Al (hν = 1486.6 eV) anode. The measurements were performed at 200 W and take-off angle of 45°. The binding energies were calibrated relative to C1s (284.6 eV) from adventitious contamination on the sample surface.

### 2.4. Ion Effect and Adsorption/Desorption Cycles

Natural water streams contain several ions, which may affect the adsorption process. Cornejo et al. [23] reported the removal of arsenic from natural waters by zero-valent iron assisted by solar radiation. They characterized the water contaminants of the Camarones River (Atacama Desert in northern Chile), in which the arsenic concentration ranges between 1000 and 1300 mg L^−1^. Besides arsenic, the analysis performed by them found chloride (Cl^−^), sulfates (${\mathrm{SO}}_{4}^{2-}$), and carbonates (${\mathrm{CO}}_{3}^{2-}$), with maximum concentrations of 670, 235, and 420 mg L^−1^, respectively. Thus, the influence of these three co-existing anions on arsenic adsorption was studied, also considering ${\mathrm{NO}}_{3}^{-}$ as a common ion present in natural waters [24]. The tests were conducted using the coexisting amounts of anions in natural waters using the batch experiment methodology described in Section 2.3. To prepare the solutions, different sodium salts (NaNO_3_, NaCl, NaCO_3,_ and NaSO_4_, over 99% pure, Merck, Darmstadt, Germany) were used. Each one was added in concentrations of 100, 250, and 500 mg L^−1^ to solutions of As (III) with 15 mg L^−1^ initial concentration and 1 g L^−1^ of CTO at pH 3.

Additionally, adsorption/desorption cycles were performed using the same batch adsorption conditions. Desorption was in a solution of NaOH 1 M for 1 h at 300 rpm, afterwards a 2 mL aliquot was taken to measure the total As release from the CTO surface. Then, the solution was centrifuged and washed with deionized water several times until neutral pH was reached. The residual solid was dried at 115 °C for 3 h and reused for the adsorption experiments up to four times. In this experiment, the total As measurement was carried out in an atomic adsorption equipment with graphite furnace brand Shimadzu model AA-6300. XRD analysis of CTO powders was also performed before and after the tests in an X-ray diffractometer PANalitycal model AERIS (Malvern Panalytical Ltda., Almedo, The Netherlands) operated at 40 Kv and 15 mA.

## 3. Results

### 3.1. Characterization of CTO Nanopowders

**Figure 2:**
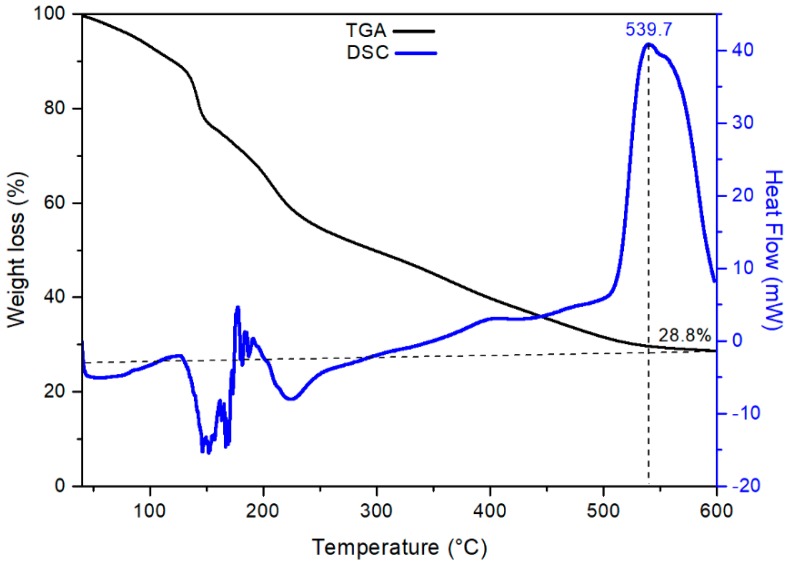
TG–DSC curves of dry gel (xerogel) powder precursor of CTO.

Figure 2 shows the TG–DSC curves of the dried precursor (xerogel). It can be seen that in the range 30 to 200 °C there is a weight loss of 42% in the TG curve (black line), which is attributed to the removal of water adsorbed on the powders surface [25]. Up to 500 °C there is an additional mass loss of 30% that would be related to the decomposition of citric acid mainly in the form of CO_2_ and NH_3_ [26]. The DSC curve (blue line) shows two endothermic peaks between 100 and 200 °C assigned to the elimination of alcohol and water [27]. Between 500 and 600 °C an exothermic peak appears attributed to the combustion of organic waste and to the crystallization process [28,29]. Based on the TG–DSC analysis, a calcination temperature of 600 °C was chosen as the optimum temperature for the formation of CaTiO_3_.

**Figure 3:**
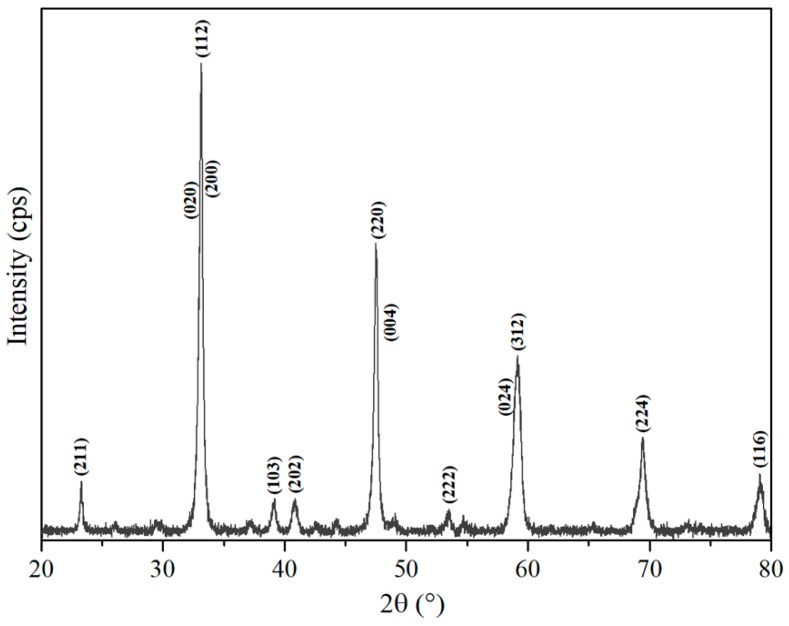
XRD pattern of CTO calcined powders.

Figure 3 displays the XRD pattern of calcined powders, which shows the formation of the single-phase CTO in the powders after the calcination step. The observed peaks in the pattern can be indexed as CTO according to the JCPDS card N° 78-1013 that corresponds to the characteristic orthorhombic phase with Pbnm space group.

**Figure 4:**
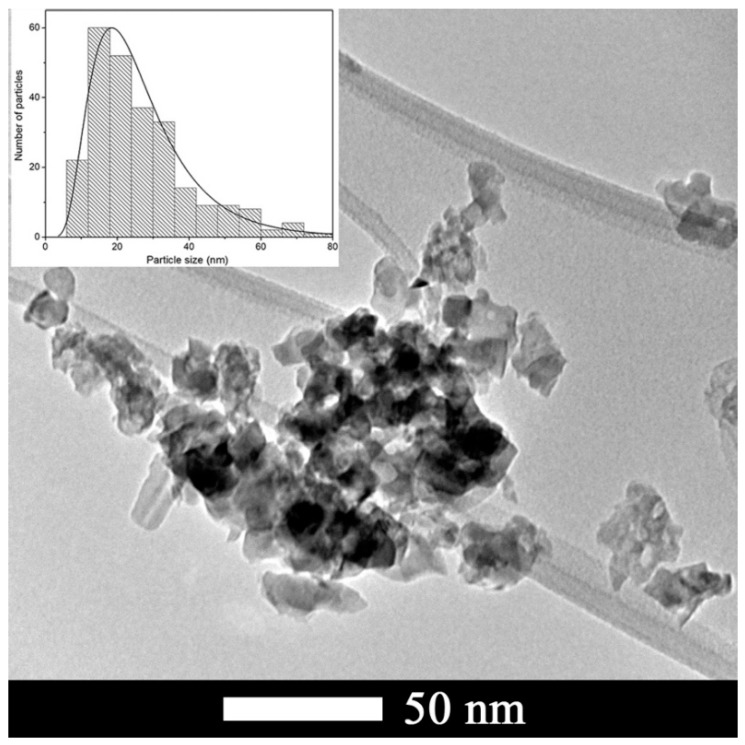
TEM image of CTO nanoparticles. Inset: Particle size distribution of 253 nanoparticles obtained from 26 TEM different images.

The morphology and microstructure of calcined CTO powders were analyzed by TEM (Figure 4). The CTO particles exhibited an irregular shape, which size ranges between 10 and 40 nm, and an average diameter of 27 nm. The inset plot of Figure 4 displays the histogram of the particles size distribution, which follows a lognormal size distribution. Elemental analysis of the CTO nanoparticles showed the presence of Ca, Ti, and O in the expected stoichiometric amounts (not shown here).

The surface specific area of CTO powders obtained by BET isotherm was 43.9 ± 0.1 m^2^/g, that is higher than the 1.6 m^2^/g of commercially CTO used by Jia et al. [30]. Zhuang et al. [31] reported surface areas between 28.34 and 108.14 m^2^/g for CTO prepared by the hydrothermal method using different titanium precursors (TiCl_4_, Ti(OC_3_H_7_)_4_ and Ti(OC_4_H_9_)_4_). Their nanoparticles exhibited different morphologies with varied particle sizes up to the nanometric range.

**Figure 5:**
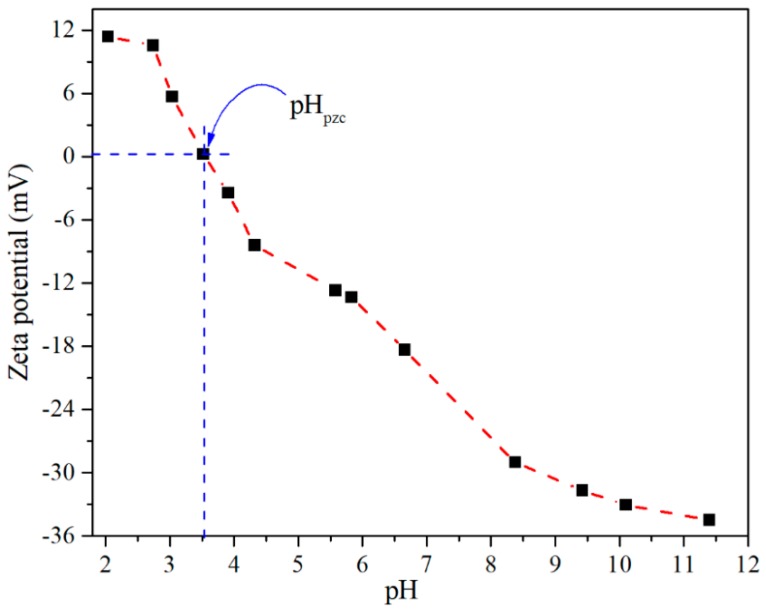
Zeta potential of CTO at pH between 2 and 11.

The Zeta Potential measurements as a function of suspended CTO were performed to determine the zero charge point or also known as the isoelectric point (IEP). This is an important parameter for the adsorption processes of any ionic adsorbate from water, since pH affects speciation of the ionic species as well as the characteristics of the absorbents surface. Based on Figure 5, the pH dependence of the zeta potential curve allows determination of the zero-charge point for CTO at pH 3.5. This value is closed to that obtained by Coreño et al. [32] where the CTO (commercial) reaches the zero-load point at pH 3.

### 3.2. Batch Adsorption Experiments

**Figure 6:**
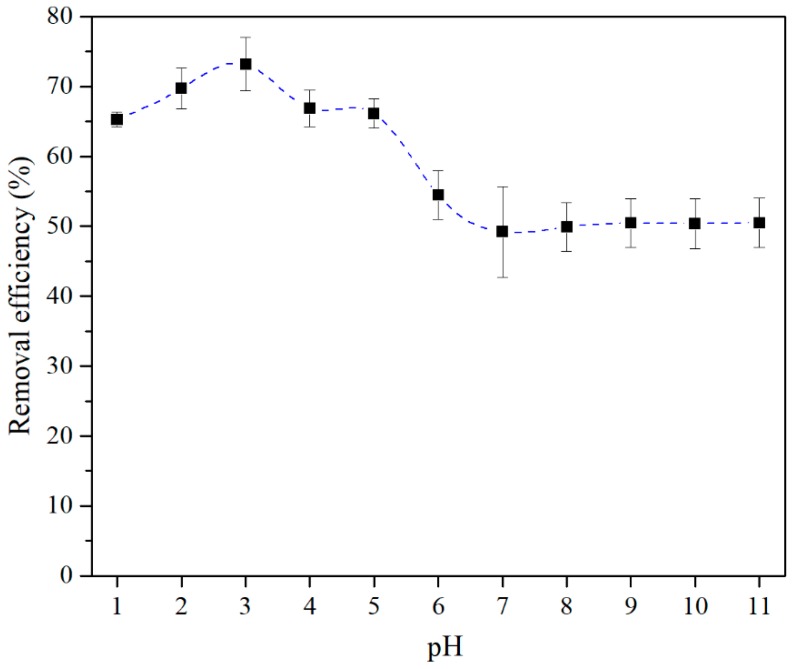
Effect of pH on As(III) adsorption by CTO nanoparticles.

Figure 6 shows the effect of pH on the adsorption of As(III) by CTO nanoparticles. The maximum value is obtained at pH 3 and, as the pH increases, the adsorption decreases until pH 7 where it reaches a plateau of constant adsorption. These results are similar to those obtained by Liu et al. [21] for Fe–Mn–Ce ternary oxide–biochar composites. In the range between pH 4 and pH 5 there is also a slight plateau, whereas at pH below 3 the adsorption tends to decrease. From the Pourbaix diagram, at low pH, neutral species (H_3_AsO_3_) predominate while over pH 7 approximately equimolar mixtures of (H_3_AsO_3_) and (H_2_AsO_3_)^−^ are present [33]. The decrease of the As(III) adsorption at pH 7 suggests that an electrostatic factor predominates due to repulsion between the negatively charge CTO surface and the oxoanion AsO(OH)^2−^. This would lead to the formation of surface complexes according to Duta et al. [33] which saturate the adsorption surface.

In contrast, below pH 5, it seems that the electrostatic ion-exchange interaction (or outer-sphere) would not explain the maximum adsorption at pH 3, at which the CTO surface becomes positive but the predominant species is neutral. Thus, the behavior at the low pH range is more likely to be related to the inner-sphere surface complex formation, i.e., As(III)–O–Ti(CTO) bonding. Wei et al. [34] proposed a microcosmic process mainly between arsenic and the surface, i.e., concerning the surface cations with positive charge which would attract the O atom of the arsenic species and the surface O with negative charge would attract the H atom of the arsenic species. This process is coherent with the fact that the maximum is near the IEP, while below pH 3, the adsorption is reduced by the high concentration of protons. The protons present in the acid solution would occupy the oxygen sites on the CTO surface, thus reducing the active sites for the adsorption of the neutral oxianion (H_3_AsO_3_) near pH 1.

**Figure 7:**
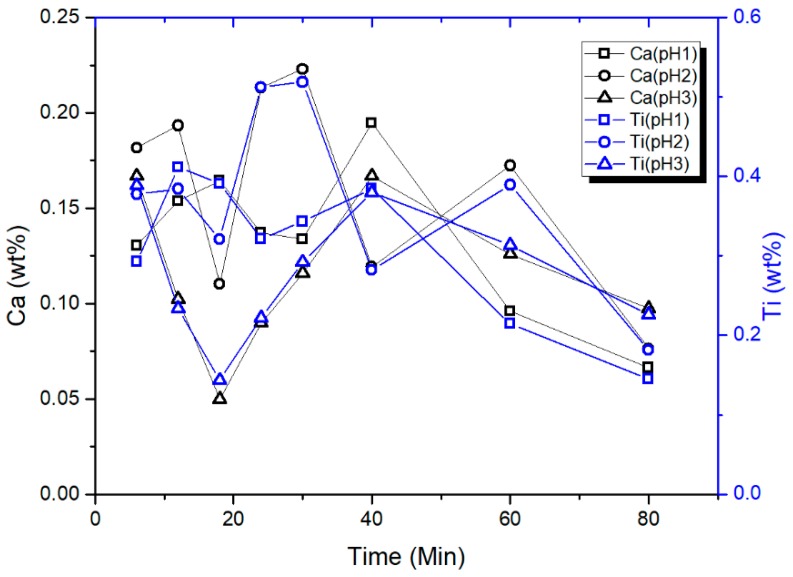
Ca (left axis) and Ti (right axis) content from aliquots obtained at different times in batch experiments, at pH 1, 2, and 3.

CTO is a very stable material and virtually insoluble at room temperature and neutral pH [35], which can also be extended to alkaline media [36]. On the other hand, in acid media, thermodynamic considerations indicate that CTO could suffer calcium leaching from the outermost layers of the particles [36]. XRF measurements to aliquots obtained at different times from batch experiments under acidic conditions, demonstrated that a residual amount of Ca and Ti are present, as is shown in the plot of Figure 7. The quantities detected do not show a correlation with the adsorption time and can be considered nearly constant. This is not consistent with a dissolution process and, on the contrary, would be attributed to residual nanoparticles that were not effectively separated during the centrifugation of the aliquots, given that the quantities detected for both elements follow the same tendency for the three pH conditions.

**Figure 8:**
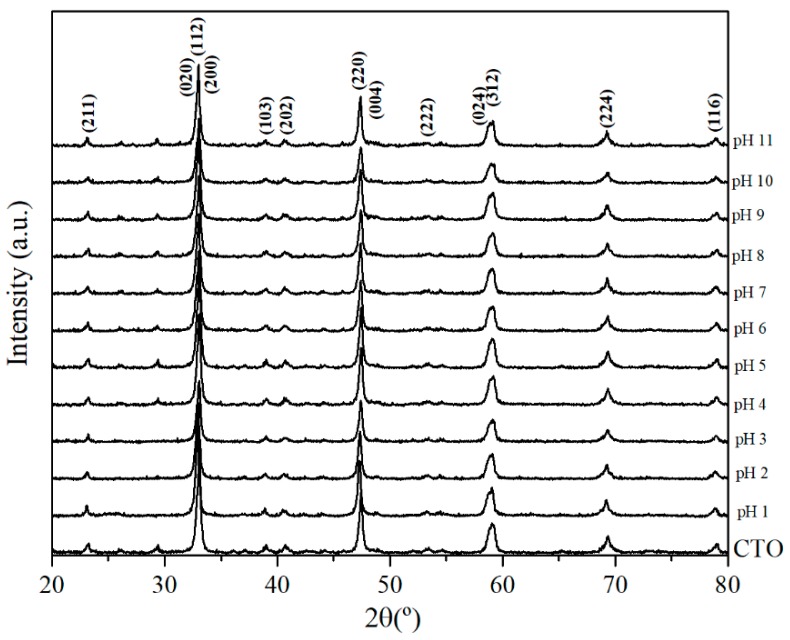
XRD patterns of CTO nanoparticles after removal of As(III) in acidic and basic conditions.

XRD analysis performed on the CTO powders after the adsorption experiments Figure 8, showed no degradation of the crystalline structure after exposition to a wide pH range. This indicates the chemical stability of CTO nanopowders under acidic and basic conditions and confirms the results discussed above.

**Figure 9:**
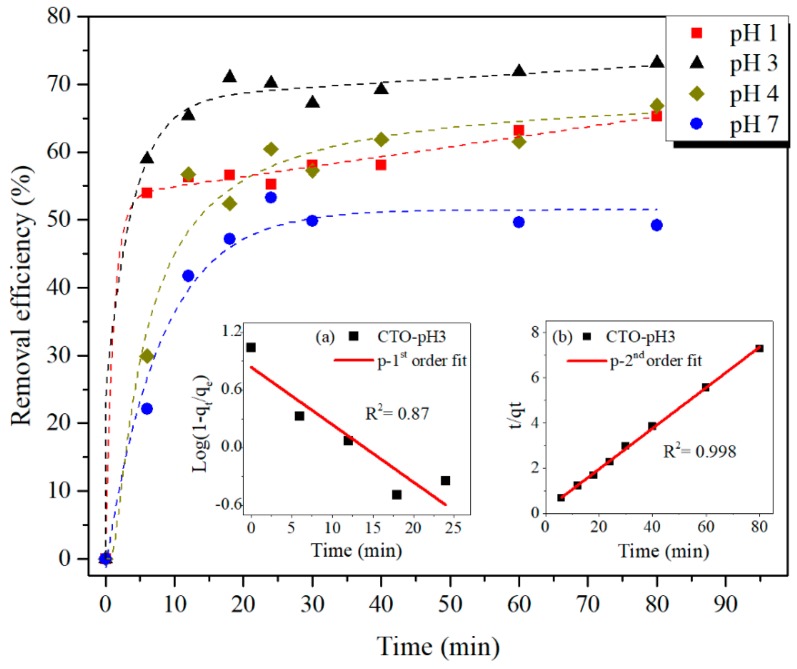
Time dependent adsorption behavior of As(III) by CTO nanoparticles at different pHs. For the sake of clarity, the curves of pH 2 and 5 are not included. Inset: Linear fit of the kinetic adsorption at pH 3 by (**a**) pseudo-first and (**b**) pseudo-second order models.

Time dependent plots of the adsorption behavior of CTO nanoparticles, measured at different pHs, are shown in Figure 9. The highest As(III) adsorption is reached at pH 3 compared to those at pH 1, 4, and 7. These results are coherent with those obtained at different pHs (Figure 6).

The adsorption curves of As(III) were analyzed using kinetic models of pseudo-first (Equation (1)) and pseudo-second (Equation (2)) order proposed by Lagergren, commonly considered for adsorption studies [37]:

(1) $$\frac{dq_{t}}{dt}=k_{1}\left(q_{e}-q_{t}\right)$$

(2) $$\frac{dq_{t}}{dt}=k_{2}{\left(q_{e}-q_{t}\right)}^{2}$$

where $q_{e}$ is the adsorption capacity at equilibrium, $q_{t}$ is the removal capacity as function of time t, $k_{1}$ and $k_{2}$ are constants of velocity of pseudo-first and pseudo-second order, respectively. The linear form of these kinetic models is expressed in Equations (3) and (4) after integrating Equations (1) and (2), respectively.

(3) $$log\frac{\left(q_{e}-q_{t}\right)}{q_{e}}=-\frac{k_{1}}{2.303}t$$

(4) $$\frac{t}{q_{t}}=\frac{1}{k_{2}q_{e}^{2}}+\frac{t}{q_{e}}$$

As an example, the insets of Figure 9 depict the fits of kinetic adsorption results at pH 3, using both models. It can be clearly seen, best fitting is obtained with the pseudo-second order model, that renders a goodness of the fit parameter (R^2^) of 0.998, higher than the R^2^ of the pseudo-first order model (R^2^ = 0.87).

The values of *k*_2_, $q_{e}$, and R^2^ of the pseudo-second order model for the different pHs are listed in Table 1. It can be verified that the highest velocity and adsorption capacity is obtained by carrying out the As adsorption at pH 3.

For similar As adsorption experiments, higher *k*_2_ values with different absorbent materials have been reported. Li et al. [38] studied the adsorption mechanism of TiO_2_-anatase that exhibited a *k*_2_ of 0.244 (mg g^−1^ min^−1^), while the $q_{e}$ (adsorption capacity in equilibrium) was of 2.639 (mg g^−1^). Similar results were shown by Pena et al. [39] for TiO_2_ nanocrystals in the adsorption of As(V and III) and, by Li et al. [20] who studied the adsorption of As by mesoporous activated carbon. In this last case, the kinetic model showed constants of *k*_2_ = 0.1866 (g mg^−1^ min^−1^) and *q_e_* = 0.425 (mg g^−1^). On the other hand, smaller *k*_2_ values, below 0.01 mg g^−1^ min^−1^, were shown by Yang et al. [40] in the adsorption of As(III) by ZnO microtubes; and also in adsorption experiments at similar initial concentrations of Mn@FeO_x_ composites [41]. It is worth noting that for all of these cases, the results of this study with CTO showed higher $q_{e}$ even at pH 7.

### 3.3. Effect of Coexisting Anions

**Figure 10:**
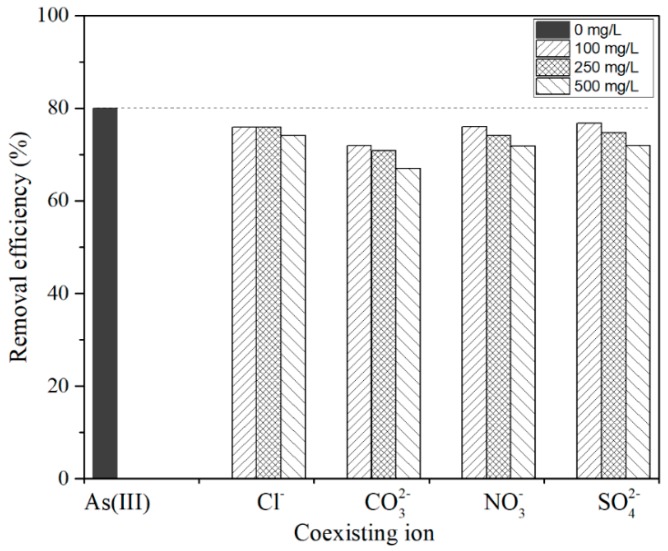
Effect of co-existing anions on As(III) removal by CTO nanoparticles (initial concentration 15 mg L^−1^, dose 1 g L^−1^, shaking 300 rpm for 2 h at room temperature and pH 3).

The influence of co-existing anions on arsenic adsorption was analyzed considering different amounts of chloride (Cl^−^), sulfates (${\mathrm{SO}}_{4}^{2-}$), and carbonates (${\mathrm{CO}}_{3}^{2-}$) and nitrates (${\mathrm{NO}}_{3}^{-}$). Figure 10 shows that an increase in the ions’ concentration leads to a decrease in the removal efficiency of As for all the coexisting elements. The stronger effect is observed for (${\mathrm{CO}}_{3}^{2-}$) that reaches a removal loss of up to 12.8% at its highest concentration (500 mg L^−1^), while the smaller influence is for (Cl^−^). This can be attributed to the higher negative charge of carbonates in comparison to the other ions, which could form complexes on the CTO surface that competes with As adsorption.

Su et al. [42] studied the removal of arsenic with porous Fe_2_O_3_–TiO_2_ ceramics. They also evaluated the effect on the arsenic removal of different ions phosphates, sulfates, and carbonates with concentrations between 10 to 50 mg L^−1^. They reported that carbonates reduce the removal of arsenic up to 50% with the highest concentration (50 mg L^−1^). In the present study, we used 10 times this concentration and a reduction four times smaller was obtained. Similarly, Thanawatpoontawee et al. [24] carried out a study on zein beads loaded with iron as a biocompatible adsorbent for the elimination of arsenic. They also evaluated the effect of common ions on arsenic removal using 100 mg L^−1^ as the maximum concentration. In their case, the ions that most affected arsenic removal were (${\mathrm{SO}}_{4}^{2-}$) and (${\mathrm{PO}}_{4}^{3-}$), decreasing by up to 50% and 80% respectively at the maximum concentration.

### 3.4. Recycle and Stability

**Figure 11:**
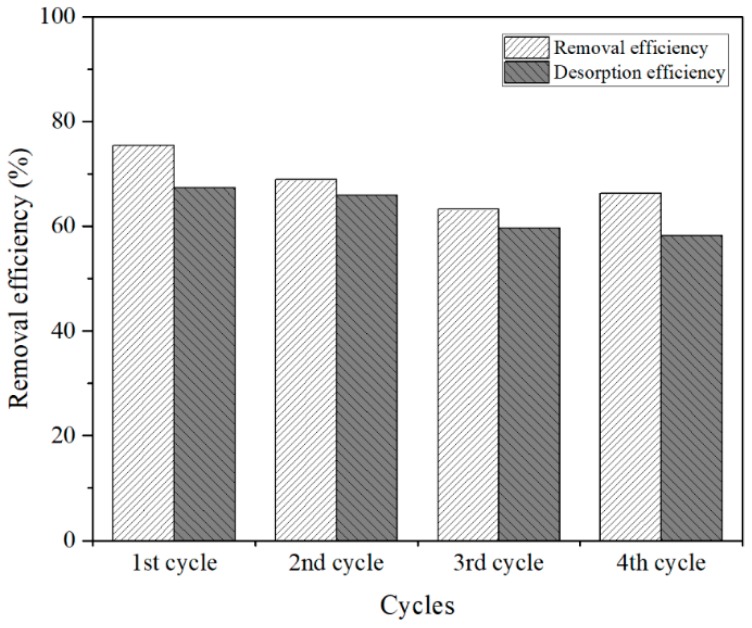
Regeneration studies of adsorption and desorption of As(III).

**Figure 12:**
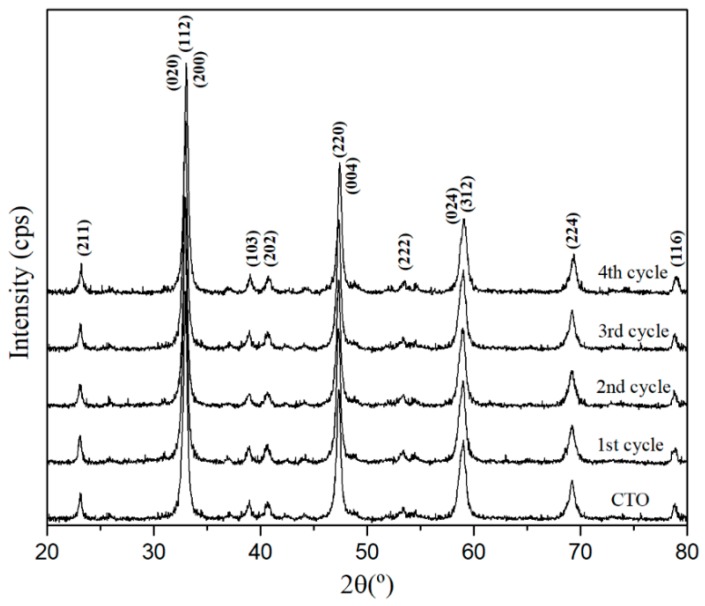
XRD pattern of CTO nanoparticles measured after each cycle of regeneration.

The recyclability or regeneration of the adsorbent material (CTO) is an important factor to minimize the treatment costs, so that the same adsorbent can be reused several times. To evaluate the recyclability of the CTO, four consecutive adsorption and desorption cycles were tested. The results are presented in Figure 11, where the adsorption results decrease to 12% in the fourth cycle and in the case of desorption, down to 17% for arsenic removal from water. Min Deg et al. [43], studied TiO_2_ nanoparticles anchored on nanosheets of Fe_3_O_4_ for the removal of arsenic. Their evaluation of regeneration exhibited a 30% drop of the arsenic removal, from the third regeneration cycle the removal of arsenic drops by 30%. The structural stability of the CTO analyzed by XRD at the end of each cycle showed that the nanoparticles were not altered by the chemical reactions during desorption–absorption cycles Figure 12.

### 3.5. Characterization of CTO Surface after Adsorption Experiments

**Figure 13:**
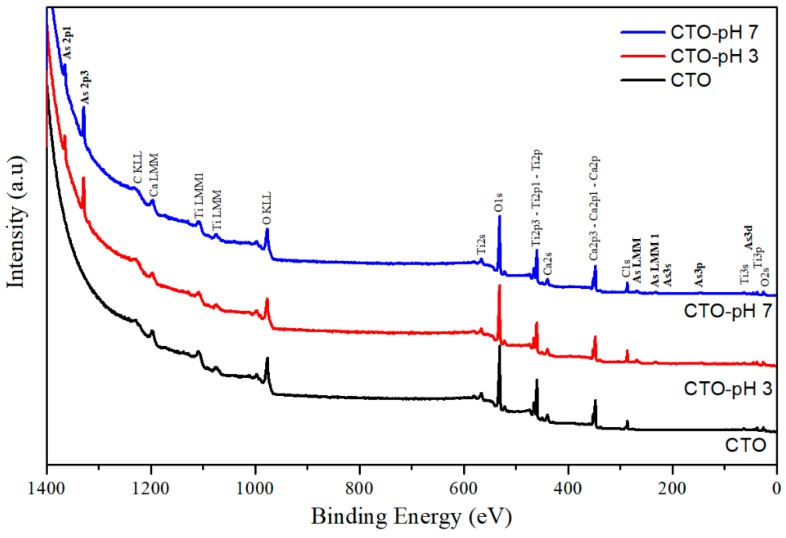
XPS survey spectra of samples before and after adsorption experiments at pH 3 and 7.

The surface of the CTO nanoparticles was analyzed by XPS after adsorption experiments to study the presence of As and its oxidation states. The samples analyzed were CTO powders before adsorption experiments, and those after adsorption kinetics at pH 3 and pH 7, CTO-pH3 and CTO-pH7, respectively. Figure 13 depicts the three spectra that show As presence in samples of pH 3 and pH 7, and the other expected signals of Ca, Ti, O, and C [44,45].

**Figure 14:**
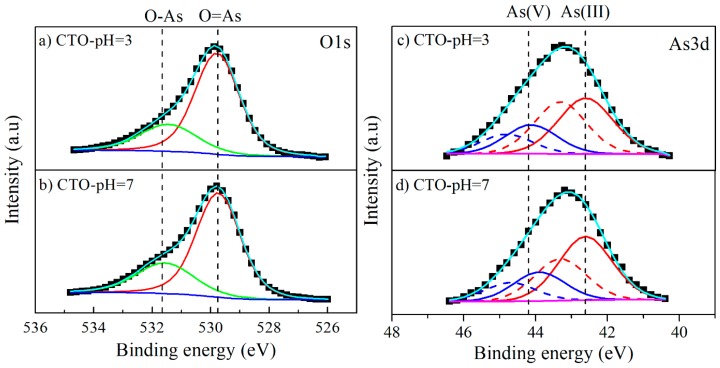
High-resolution XPS spectra of CTO after adsorption experiments at pH 3 and 7.

To elucidate the states of the different species after adsorption, high-resolution XPS spectra (HRXPS) were also obtained. The HRXPS of Ti2p and Ca2p (not included here) does not show any change on the incorporation of As on the CTO surface. On the other hand, the O1s signal in CTO after As adsorption, Figure 14a,b, exhibited two contributions from the different oxygen species: at 531.6 eV binding energy (O–As), and 529.8 eV of binding energy (O=As). A binding energy shift of these oxygen species is observed, which can be explained by the CTO presence and the O=As–O formation from the arsenite removal.

For the As 3d signal, the fit was performed considering the As3d5/2 and As3d3/2 doublets, separated by 0.69 eV and area ratio of 5/3 [46], and taking into account an energy separation between both signals of 1.4 eV [47,48]. After adsorption experiments, the CTO-pH3 sample exhibited two doublets at 42.6 and 44.1 eV (Figure 14c), identified as As(III) and As(V), respectively [46,47,48]. Sample CTO-pH7 showed similar results (Figure 14d) but a shift of the As(V) binding energy to 43.9 eV, Nevertheless, the ratio between As(III) and As(V) signals does not change by the pH increase in the solutions. Thus, XPS results indicate that As is adsorbed on the surface of CTO nanoparticles as As(III) and is partially oxidized to As(V). Similar behavior was observed by Jegadeesan et al. [49] for As sorption on TiO_2_ nanoparticles, which could be attributed to the presence of surface hydroxyl groups or physiosorbed oxygen.

## 4. Conclusions

The CTO nanoparticles prepared by the sol-gel technique were demonstrated to be a good adsorbent of As(III) with a maximum adsorption capacity of 11.12 mg g^−1^ at room temperature, which is higher than other values reported in similar studies. It was shown that the adsorption capacity of As varies with the pH of the solution, and that the maximum adsorption capacity was given at pH 3 which coincides with the isoelectric point of the CTO nanoparticles. This is related to an inner-sphere surface complex formation of As(III)–O–Ti(CTO) bonding. The study on adsorption kinetics shows that this system follows a pseudo-second order model. The X-ray photoelectron spectroscopy analysis of the CTO surface after removal experiments, showed that arsenic was present as As(III) and partially oxidized to As(V). Under these conditions of removal, the addition of interfering ions (${\mathrm{CO}}_{3}^{2-}$, ${\mathrm{NO}}_{3}^{-}$, ${\mathrm{SO}}_{4}^{2-}$ and Cl^−^) did not cause a significant decrease of As(III) adsorption using CTO. Additionally, the regeneration of the CTO is possible and confirms that the adsorption is reversible.

## Figures and Tables

**Table 1:** Calculated parameters from the second order kinetic model of the arsenic removal kinetics with CTO, in function of pH.

Pseudo-Second Order Kinetic Parameters	*k*_2_ [g mg^−1^ min^−1^]	*q_e_* [mg g^−1^]	R^2^
pH 1	0.0296	10.00	0.996
pH 2	0.0374	10.43	0.994
pH 3	0.0508	11.12	0.999
pH 4	0.0406	9.65	0.996
pH 5	0.0211	8.37	0.975
pH 7	0.0250	8.89	0.974
